# Hypocholesterolemic, Antioxidative, and Anti-Inflammatory Effects of Dietary *Spirulina platensisis* Supplementation on Laying Hens Exposed to Cyclic Heat Stress

**DOI:** 10.3390/ani12202759

**Published:** 2022-10-14

**Authors:** Morshed I. M. Al-Otaibi, Hasan A. E. Abdellatif, Abdelmohsen K. A. Al-Huwail, Ahmed O. Abbas, Gamal M. K. Mehaisen, Eman S. Moustafa

**Affiliations:** 1 Department of Animal and Fish Production, College of Agricultural and Food Sciences, King Faisal University, P.O. Box 420, Al-Ahsa 31982, Saudi Arabia; 2 Department of Internal Medicine, Faculty of Medicine, Al-Azhar University, Assiut 71511, Egypt; 3 Department of Animal Production, Faculty of Agriculture, Cairo University, Giza 12613, Egypt

**Keywords:** cholesterol profile, heat stress, inflammatory cytokines, laying hens, productive performance, redox status, *Spirulina platensis*

## Abstract

**Simple Summary:**

Heat stress remains one of the critical environmental conflicts in the field of the poultry industry. Recently, dietary modifications with various natural products have been used as a potential strategy to relieve the various deleterious impacts of heat stress on poultry flocks. This study was proposed to investigate the beneficial impacts of different dietary levels of microalgae “Spirulina platensisis” on the productive performance, cholesterol profile, redox status, and pro-inflammatory cytokines in laying hens suffered from heat stress. Based on the results, the spirulina could be supplemented into the diets to improve the layer’s production of lower-cholesterol eggs and reduce the negative impacts of heat stress on the other physiological aspects.

**Abstract:**

This study aimed to investigate the role of dietary *Spirulina platensis* (SP) supplementation in relieving the negative impacts of heat stress (HS) on the productive performance, cholesterol profile, redox status, and inflammatory cytokines of laying hens. A total of 288, 45-wk-old and 1550.7 ± 2.3 g initial body weight, HY-Line W-36 laying hens were housed in two environmental-controlled compartments. Layers were allotted to eight treatments of a two × four factorial design, with six replicates containing six birds per treatment. The temperature in one of the compartments was kept at a thermoneutral condition (24 °C group), while the temperature in the other compartment was raised to a cyclic heat stress of 35 °C from 9:00 a.m. to 5.00 p.m. (35 °C group). Layers in each compartment were fed on one of four experimental diets, containing 0%, 3%, 6%, or 9% SP (SP groups). The trial continued for five weeks. As a result of this study, exposure of laying hens to cyclic HS resulted in a significant (*p* < 0.05) increase in the total cholesterol (CH), low-density lipoprotein-CH, liver- and egg yolk-CH, ceruloplasmin, malondialdehyde, interleukins (IL-1β and IL-6), and tumor necrosis factor-α, and a significant (*p* < 0.05) decrease in the high-density lipoprotein-CH, total antioxidant capacity, and reduced glutathione levels. HS negatively (*p* < 0.05) affected the hen–day egg production (EP, 90.5% vs. 77.0%), egg weight (EW, 61.8 g vs. 56.8 g), feed intake (FI, 111.6 g vs. 101.5 g) and feed conversion ratio (FCR, 2.00 vs. 2.37). As SP levels increased in layer diets, a linear (*p* < 0.05) improvement response in most of the parameters was obtained in both HS and non-HS layers, recording the best results with 9% SP (e.g., 78.8% vs. 87.6% EP, 56.7 g vs. 61.9 g EW, 103.3 g vs. 110.2 g FI, and 2.38 vs. 2.04 FCR, in 0% vs. 9% SP, respectively). When incorporating SP into the diets of HS-layers, the negative impacts of HS were remarkably relieved (*p* < 0.05). Therefore, diets containing 9% SP could be used as a promising approach to improve the productive and physiological performance of laying hens, particularly under heat stress conditions.

## 1. Introduction

In some arid regions, exposure of poultry flocks to the hot climate leads to a huge economic drop in the business sector of egg production [1,2]. Heat stress (HS) is a case of imbalance in body thermoregulation and physiological homeostasis of laying hens which dramatically affects their productive performance [3,4]. The reports talked about substantial decrease in egg productivity and feed efficiency of laying hens when affected by acute or chronic HS [4,5]. It was reported that these events likely resulted from the reduction in feed consumption, diet digestion, gut integrity, and protein synthesis in HS-chickens [4,6,7].

It has been documented that HS may affect some biological activities in poultry species [8], inducing a disturbance in the redox system [3,9], or an elevation in the pro-inflammatory cytokines [2,5,10]. Previous studies also demonstrated that HS inhibited the function of antioxidant enzymes, such as superoxide dismutase and glutathione peroxidase, and non-enzymatic antioxidants, such as glutathione and vitamins [3,11]. In a recent study, Abbas et al. [12] found that layers suffering from cyclic HS displayed high levels of plasma corticosterone, lipid peroxidation, interleukin-1β and tumor necrosis factor-α compared to non-HS layers. Furthermore, it was revealed that HS increased the levels of cholesterol in broiler’s serum from 104 mg/dL in thermoneutral groups to 152 mg/dL in HS groups [13]. It was also shown that HS up-regulated the hepatic expression of lipogenic proteins and increased the liver triglyceride content, the plasma low-lipoprotein and total cholesterol levels in HS-broiler chickens [14,15,16].

Dietary modifications have been used as a potential strategy to relieve the various deleterious impacts of HS on laying hens [12,17,18,19], broilers [14,20,21,22,23,24,25,26,27], and other poultry [10,28,29,30,31]. One of the potential natural resources that can be suggested in poultry nutrition for this purpose is *Spirulina platensisis* (SP). These blue-green microalgae are known to have high contents of protein, amino acids, fatty acids, vitamins, minerals, carotenoids, and other fundamental nutrients [32,33]. In addition to its nutritional value, SP possesses paramount biological properties such as immunomodulation [34,35], tumor resistance [36], anti-inflammation [37], anti-hyperlipidemia [38], and antioxidant activity [39]. It was found that dietary supplementation with 0.5–2 g/kg of the powder form of SP enhanced the productive performance and physiological responses, including lipid profile, redox status, humoral immunity, blood metabolites, and hematological parameters, of broiler chickens exposed to cyclic heat stress [40,41]. It was also reported that SP can potentially reduce the HS-biomarkers such as lipid peroxidation and heterophil to lymphocyte ratio in laying quail birds suffering from HS [42].

To our knowledge, there is not enough studies discussing the possible effects of SP inclusion into layer diets on their productive outputs, especially under heat stress conditions. Determination of an appropriate level for SP application and interpretation of SP physiological effects during heat stress need to be intensively investigated. Thus, the goal of this study was to highlight the potential role of inserting various levels of SP into laying hens’ diets to ameliorate the negative impact of HS on productivity, cholesterol profile, redox status, and inflammatory biomarkers.

## 2. Materials and Methods

### 2.1. SP-Microalgea Analysis

A freeze-dried SP powder was purchased from a commercial supplier (Inner Mongolia Rejuve Biotech. Co., Ltd., Ordos, China). The SP powder was stored at room temperature until use in experiments. The chemical composition of the SP was analyzed according to the guidelines of the Association of Official Analytical Chemists (AOAC) [43]. The total polyphenols and flavonoids of the SP were assessed by the Folin–Ciocalteu method and the aluminum chloride calorimetric method, respectively [44]. Gallic acid equivalents (GAE) were used as a standard curve for the quantification of phenolic contents per g SP. Quercetin was used to generate a standard curve to deduce the flavonoid concentrations as quercetin equivalents (QE) per g SP. In addition, the total antioxidant activity was determined by the radical-scavenging 2,2-diphenyl-1-picrylhydrazyl (DPPH) test following the procedure described by Moukette Moukette et al. [45]. The results of SP chemical composition analysis are presented in Table 1.

### 2.2. Birds and Management

Forty-four-week-old HY-Line W-36 commercial laying hens of 1550.7 ± 2.3 g initial body weight (288 layers in total) were housed in two compartments with environmental controlled systems. The hens were maintained individually in 40 × 40 × 50 cm cages. For one week, the compartments were provided with a temperature of approximately 24 ± 1 °C, relative humidity of 50% and 30 Lux LED lighting of 17 h/day. The cages were supplied with a stainless-steel feeder and a nipple drinker. All diets were mashed, with feed and water provided ad libitum during the study. Birds were monitored twice during the application of heat stress to detect any signs of suffering such as breathing difficulty, watery discharge, decreased appetite, and ruffled feathers. If such signs appeared, euthanasia was allowed by cervical dislocation to prevent pain from the stress.

### 2.3. Experimental Design and Sample Collection

From 45–50 weeks of age, layers were allotted to 8 treatments according to a 2 × 4 factorial design, with six replicates containing six birds per treatment. The temperature in one of the compartments was kept at a thermoneutral condition (24 °C group), while the temperature in the other compartment was raised to a cyclic heat stress of 35 °C from 9:00 a.m. to 5.00 p.m. then reduced outside of these times to the neutral temperature (35 °C group). Layers in each compartment were fed on one of four experimental diets, according to the SP level (SP groups), included with 0%, 3%, 6%, or 9% SP. The experimental diets were formulated based on the nutritional guidelines of the commercial HY-Line W-36 layers [46] (Table 2), and the chemical analysis was determined using the methods described by the AOAC [43]. Eggs were collected and weighed daily to assess the average hen–day egg production (EP%) and egg weight (EW) during the entire experiment. The feed intake (FI) was recorded daily. Feed conversion ratio (FCR) was then calculated based on the total FI per egg mass produced per hen. As soon as the trial ended, 3 eggs were harvested from each replicate and broken to separate the yolks. Three hens per replicate (*n* = 18) were slaughtered by cervical dislocation to obtain liver specimens. The egg yolks and livers were assigned to determine the cholesterol levels. In addition, 3 hens per replicate (*n* = 18) were bled quickly within 3 min during the night to avoid handling stress as much as possible [47]. Blood samples were centrifuged at 2000× *g* for 10 min at 4 °C, and then the plasma was separated for the cholesterol in plasma, the redox, and the inflammatory biomarkers. The plasma, yolk and liver samples were stored in liquid nitrogen (LN_2_) until further analysis. A scheme for the experimental design is presented in Figure S1.

### 2.4. Cholesterol Profile

The egg yolk and liver cholesterols (CH), as well as the total plasma CH, high-density lipoprotein CH (HDL-CH), and low-density lipoprotein CH (LDL-CH) were quantified according to the Abcam kit’s protocol (ab65390, Cambridge, MA, USA), according to methods described in a previous work [48]. Briefly, all samples were thawed in an ice bath. Plasma samples were used directly after thawing to detect the total-CH levels. To separate the HDL- and LDL-CH from the plasma, a mixture of 100 μL of the plasma and 100 μL of 2× precipitation buffer was centrifuged twice at room temperature at 2000× *g* for 10 min. The HDL-CH fraction was aspirated carefully with the supernatant, while the precipitate was resuspended in 200 μL PBS to obtain the LDL-CH fraction. The yolk and liver samples were first homogenized by mixing 10 mg with 100 μL of cholesterol assay buffer into an appropriate pestle sitting on ice with approximately 10–15 passes. The homogenate was cold centrifuged for 5 min at 13,000× *g*, and then the supernatant was collected for analysis. Microplate wells were filled with 50 μL of the CH-working standard solutions or the sample, then 50 μL of total CH reaction mix was added to all wells. After incubation at 37 °C for 60 min in the dark, the microplates were read immediately at optical density (OD) 570 nm using a microplate reader (ELx808™, BioTek Instruments, Winooski, VT, USA). The CH level in the test samples was calculated as (A/V × D × 100); where: A = amount of cholesterol in the sample well calculated from standard curve (μg), V = sample volume added in the sample wells (μL), and D = 1 for total-CH or 2 for HDL- and LDL-CH fractions.

### 2.5. Redox Status Analysis

The redox status was evaluated by measuring the plasma levels of ceruloplasmin (CP), malondialdehyde (MDA), total antioxidant capacity (TAOC), and reduced glutathione (GSH). Plasma CP was determined using chicken ELISA kits (MBS1609488, MyBioSource Inc., San Diego, CA, USA), according to methods described by Song et al. [49]. The plasma MDA, TAOC and GSH were determined using colorimetric assay kits (E-BC-K025-S, E-BC-K136-S, and E-BC-K030-M, respectively; Elabscience Biotechnology Inc., Houston, TX, USA). The MDA and GSH analyses were performed according to Moustafa et al. [41], while the TAOC assay was performed according to Uwikor et al. [50].

#### 2.5.1. Plasma CP assay

In brief, 100 μL of the standard or the plasma was pipetted into pre-coated microplate wells and incubated for 2 h at 37 °C. The wells were washed three times, then 100 μL of biotin-conjugate was added to each well and incubated for 1 h at 37 °C. the wells were washed again 3 times, refilled with 100 μL of streptavidin- horseradish peroxidase (HRP) and incubated for a further 1 h at 37 °C, followed by 5 washes. One hundred μL of substrate solution was added to the wells and incubated for 20 min at 37 °C in the dark. After that, 50 μL of stop solution was added and the raised color was measured at 450 nm using a microplate reader. The intra-assay and inter-assay coefficients of variability (CV) were <8% and <12%, respectively, and the detection range was 10–2000 ng/mL.

#### 2.5.2. Plasma MDA Assay

Four tubes were filled with 100 μL of either absolute ethanol (blank tube), standard solution (10 nM/mL, standard tube), or two volumes of the plasma (control and sample tubes) and were mixed with 100 μL of clarification reagent 1 and 3 mL of acid reagent 2. One mL of chromogenic reagent 3 was added to the blank, standard and sample tubes, while the control tube was mixed with 1 mL of 50% glacial acetic acid. The tubes were incubated at 95–100 °C for 40 min, then cooled to room temperature with running water, and centrifuged at 3100× *g* for 10 min. The supernatant was read with a 1 cm optical path cuvette at 532 nm using a spectrophotometer (CE1010, Cecil Instruments Limited, Cambridge, UK). MDA content (nmol/mL) was calculated as (ΔA_1_/ΔA_2_ × C), where ΔA_1_ = OD_sample_ − OD_control_, ΔA_2_ = OD_standard_ − OD_blank_, and C = standard concentration. The intra-assay and inter-assay CV were 4.9% and 8%, respectively, and the detection range was 0.38–133.33 nM/mL.

#### 2.5.3. Plasma TAOC Assay

One hundred μL of the plasma was put into the sample tube containing 1 mL of buffer reagent solution, then mixed with 2 mL of chromogenic reagent and 0.5 mL of ferric salt reagent. The tubes were incubated at 37 °C for 30 min followed by addition of 100 μL of stop solution reagent. Thereafter, 100 μL of the plasma was added to the control tube. After 10 min at room temperature, the OD was obtained by the spectrophotometer at 520 nm. TAOC was measured as units per mL according to the equation [ΔA/(0.01 × 30) × V_1_/V_2_), where ΔA = OD_sample_ − OD_control_, 0.01 = the OD increase per min, 30 = the reaction time min, V_1_ = the total volume of reaction, and V_2_ = the sample volume. The intra-assay and inter-assay CV were 2.7% and 8.2%, respectively, and the detection range was 0.62–145.2 U/mL.

#### 2.5.4. Plasma GSH Assay

One hundred μL of the plasma was mixed with 100 μL of reagent 1 and centrifuged at 4500× *g* for 10 min. The microplate wells supplemented with 25 μL of reagent 3 were filled with 100 μL of the sample supernatant (sample wells), 100 μL of reagent 1 (control well), or 100 μL of the standard dilutions (standard wells). After that, 100 μL of reagent 2 was added to all wells and maintained for 5 min at room temperature. The OD values for standards (OD_st_), samples (OD_sp_) control (OD_c_) and blank (OD_b_ = OD of zero standard) were obtained at 405 nm using a microplate reader. The slope (a) and intercept (b) of the standard curve were obtained using graph software (y = ax + b); where y = OD_st_ − OD_st0_, and x = standard concentration. The GSH content (nM/mL) was then calculated using the equation: [(OD_sp_ − OD_c_ − b)/a × 2]. The intra-assay and inter-assay CV were 1.9% and 3.2%, respectively, and the detection range was 2–100 µM/mL.

### 2.6. Inflammatory Cytokine Analysis

The inflammatory cytokines were evaluated by measuring the plasma levels of interleukin-1 beta (IL-1β), interleukin-6 (IL-6), and tumor necrosis factor-alpha (TNF-α). ELISA kits specific for chickens were obtained from MyBioSource Inc. (MBS2024496, MBS2021018, and MBS2031870, respectively) and the protocol guidelines were followed for each analysis. In summary, 100 μL each of standard dilutions and the plasma samples were added to the appropriate antibody-precoated microplate wells and incubated at 37 °C for 1–2 h according to the analysis. After removing the liquid from the wells, 100 μL of prepared detection reagent A was added, followed by an incubation at 37 °C for 1 h. The wells were washed three times, then filled with 100 μL prepared detection reagent B followed by further incubation for 30 min at 37 °C. After washing 5 times, 90 μL of the substrate solution was added to the wells and incubated for 20 min at 37 °C. Thereafter, 50 μL of the stop solution was added to the wells and the microplate was immediately read at 450 nm. The intra-assay and inter-assay coefficients of variability (CV) were <10% and <12% respectively, for all assays. The detection range IL-1β, IL-6, and TNF-α were 15.6–1000 pg/mL, 7.8–500 pg/mL, and 7.8–500 pg/mL, respectively.

### 2.7. Statistical Analysis

Data were arranged following 2 × 4 factorial design and analyzed with a multivariate test of a general linear model (GLM) using the IBM SPSS Statistics version 22 (IBM Corp., Armonk, NY, USA) [51]. A polynomial contrast analysis was done to check the linear and quadratic effects for the increasing SP levels on all variables. The group means, standard error of means (SEM), and *p*-values were shown for the main effects of HS (24 °C versus 35 °C), SP levels (0%, 3%, 6%, and 9%), and their interaction (HS × SP). The differences between the group means were tested using “Tukey’s post hoc” test. Statistical significance was considered to exist at *p*-value < 0.05. The data were examined for a normal distribution before performing the statistical analysis.

## 3. Results

### 3.1. Cholesterol Profile

The effects of HS, SP levels and their interaction on layer cholesterol profiles are presented in Table 3. Data analysis showed a significant (*p* < 0.05) increase in the plasma total-CH, LDL-CH, and egg yolk- and liver-CH; in contrast, the plasma HDL-CH showed a significant decrease. The SP treatment showed a linear decrease (*p* < 0.05) in the plasma total-CH, LDL-CH, and egg yolk- and liver-CH as the SP level increased. The HDL-CH increased linearly as the SP level increased (*p* < 0.05). Moreover, the SP supplementation in HS-layers ameliorated (*p* < 0.05) the elevation in deleterious cholesterols (total-CH, LDL-CH, yolk-CH), and ameliorated the reduction in the HDL-CH by heat stress. There was no significant effect for HS × SP interaction on the liver-CH (*p* > 0.05).

### 3.2. Redox Status

The effects of HS and SP levels and their interaction on layer redox status are shown in Table 4. HS significantly (*p* < 0.05) increased the CP and MDA levels in plasma, while it showed a significant decrease in the TAOC and GSH, plasma HDL-CH. On the contrary, the CP and MDA levels decreased linearly and the TAOC and GSH increased linearly as the dietary SP level increased (*p* < 0.05). Further, there was a significant interaction between HS and SP treatments in all redox parameters (*p* < 0.05). The SP supplementation in both HS and non-HS groups improved the redox status of layers compared to non-supplemented groups. In addition, the lowest CP and MDA levels in the HS-layers were obtained when SP was added to the diets at 9% compared to the other levels in the same group.

### 3.3. Inflammatory Cytokines

The effects of HS, SP levels and their interaction on layer inflammatory cytokines are presented in Table 5. Results indicated that HS significantly (*p* < 0.05) increased the IL-1β, IL-6, and TNF-α. On the contrary, the IL-1β, IL-6 and TNF-α levels were decreased linearly (*p* < 0.05) by the increase in dietary SP levels. Moreover, the supplementation of SP significantly (*p* < 0.05) alleviated the increase in inflammatory cytokines in the HS-layers, showing the lowest levels of IL-1β, IL-6, and TNF-α when applying 9% SP compared to the other SP levels.

### 3.4. Layer Performance

The effects of HS, SP levels and their interaction on layer performance are illustrated in Table 6. The layer productive performance was negatively influenced by HS exposure. There was a significant (*p* < 0.05) decrease in EP by 13.5 p.p., in EW by 5.0 g, and in FI by 10.1 g in the HS-layers compared to the non-HS-layers. The FCR worsened by 18.5% in the HS-layers than in the non-HS layers. In contrast, the dietary SP treatment significantly improved the layer performance in a linear trend with the increasing level of SP (*p* < 0.05). Under heat stress conditions, dietary SP supplementation at the level of 9% significantly increased the EP by 13.8 p.p., the EW by 9.1 g and the FI by 4.9 g, while it decreased the FCR by 34.8%, respectively, compared to the HS group without SP supplementation (*p* < 0.05).

## 4. Discussion

The results of the present study demonstrated the existence of the destructive effects of heat stress on laying hen’s productive performance. Several studies reviewed the side effects of heat stress exposure on poultry to be numerous and significantly influence both wellbeing and productivity of the birds [52,53]. It was documented that high temperature negatively influences the process of egg formation at both ovarian and reproductive tract levels, including ovulation and oviposition [54], and reduces the feed intake, energy availability, nutrients digestibility, and metabolism [55]. Consistent with other previous research [4,9,12], a substantial depression was noticed in the EP and EW for HS-layers when compared to their control. The obvious decrease in egg quantity could be reasoned to be directly related to the depression in FI and FCR by exposure of birds to HS in the same group [1,6,7]. Other physiological alterations happened in HS-layers and might contribute to the low performance of laying hens in the present study. HS decreased antioxidants (TAC and GSH) and increased other plasma stress indicators assessed in the present study, including the pro-inflammatory cytokines (IL-1β, IL-6 and TNFα) and lipid peroxidation (MDA). It was recently suggested that HS impaired egg production by inducing follicular cell apoptosis through excess production of MDA and reactive oxygen species (ROS), and the activation of corticosterone (CORT)-induced TNFα pathways [2,56]. Furthermore, HS increased the levels of harmful cholesterols in the laying hens (Table 3). These results can be explained by the action of glucocorticoids, which are released because of the hypothalamic–pituitary–adrenal axis stimulation in HS-birds [57]. In addition, HS mediates several pathways involved in the biosynthesis of cholesterol [58]. In line with our results, it was reported that HS increased liver triglycerides and CH formation in avian liver and serum [15,16].

Spirulina has been suggested as a supplement in poultry nutrition because of its rich nutrients and biological functions [32,33]. According to the chemical analysis of SP in our study, it contained rich amounts of protein (56.4%), vital minerals such as Ca, P, Na, and K (Table 1). There was a better performance of laying hens with the SP supplementation at 90 g/kg (as fed) compared to the control group. The diets supplemented with SP also had increasingly more yellow corn, indicating a difference of approximately 25.9 g/kg (as fed) between the control and the 9%-SP groups (Table 2). Although the chemical composition of the yellow corn was not analyzed in this study, it is known from the pamphlet of the feed company supplier that yellow corn contains a maximum of 7.5% protein. It calculatedly means that 9%-SP can add 50.8 g protein to the control basal diet, while yellow corn can add only 1.9 g protein. Thus, the better performance of laying hens in the 9%-SP group could be attributed to the higher contribution of the SP compared to the other traditional ingredients to increase the protein content of the diets.

On the other hand, the positive effect of SP as an antioxidant was proved in previous studies on mammals [39,59,60] and on poultry [41,61,62]. The total polyphenols and flavonoids of SP in the present study are high enough to augment the antioxidant activity of the SP (29.2%, Table 1), and this consequently linearly improved the redox status of layers when fed on incremented levels of the SP (Table 4). Furthermore, the increased SP levels in layer diets linearly reduced the inflammation as displayed in our study (Table 5) and reported in other studies [63,64]. It was reported that phenolic compounds and flavonoids can terminate the chain reaction of ROS and similar products before seriously affecting cell viability [65]. These events, therefore, may contribute to the linear improvement response found for increasing levels of SP on the productive aspects of laying hens. Our results agree with that reported in previous studies [66,67] that laying hens fed on SP had the best means of EP, EW and FCR compared to the control group.

The interaction analysis of HS × SP treatments in the present study indicated that the addition of SP to layer diets was able to ameliorate the negative impacts of HS on the layer physiological and productive performance. Results indicated that despite the severe reduction in all productive parameters of HS-layers, the incorporation of SP (especially at 9%) alleviated the effect of HS on layer production (Table 6). On the other hand, SP treatment lowered the levels of total-CH, LDL-CH and yolk-CH again, after extreme elevation by HS, while SP increased the level of HDL-CH in the HS-layers. These results may introduce the importance of adding SP into layer diets, especially in arid hot regions, to produce healthier eggs and prevent cholesterol-induced atherosclerosis in humans [68]. Such hypocholesterolemic effect of the SP was demonstrated in previous studies on experimental rats [69], and more specifically on HS-broiler chickens [41,62]. Deng and Chow [70] explained that SP contains γ-linoleic acid which binds CH metabolites in the bile and prevents its accumulation. The SP treatment, linearly and quadratically decreased the liver-CH, but this decrease failed to reach significant levels under heat stress conditions. The positive effect of SP on liver-CH may not be employed due to the limitation of apolipoprotein B (ApoB), which is essential to transport excessive lipids from the liver to extrahepatic tissues in HS-birds [15].

Under heat stress conditions, the negative impact of HS on poultry is mostly accompanied with a disturbance in the redox status [4,53]. In contrast, the current results indicated that the elevation in MDA and CP, which are oxidative stress indicators, were again extremely reduced when SP was added to the layer diets at 9%, while the GSH and TAC were increased by SP treatment in the HS-layers. The capacity of SP to bring back the redox balance in HS-layers could be attributed to its bioactive antioxidant compounds such as flavonoids and polyphenols (Table 1) and other antagonists to free radicals such as α-tocopherol, ascorbic acid, β-carotene and selenium [71,72].

The present study showed an ameliorative action for the SP treatment on the elevated pro-inflammatory cytokines in HS-layers. However, there is little information about the mechanism of spirulina action on the inflammation indicators in poultry, and even during heat stress. Mullenix et al. [63] reported that SP supplementation alleviated the inflammation in broilers fed on low protein diets, by reducing mRNA expression of IL-6, IL-10, and other interleukins. Other trials have also indicated that algae ameliorate the molecular expression of pro-inflammatory cytokines in other challenged species [73,74]. The presence of vital minerals, such as Zn, Mn, Fe, Mg, K and Ca, in the SP may be responsible for alleviating the HS-induced inflammation and reducing the expression of some stress indicators including HSP70, IL-18, and TNF-α in the blood [75]. Also, the presence of polyphenols in the SP may promote several molecular mechanisms correlated with anti-inflammation such as inhibition of arachidonic acid enzymes, inhibition of the nuclear factor kappa B (NF-κB) pathway, activation of phase-II antioxidant detoxifying enzymes, and activation of mitogen activated protein kinase (MAPK), protein kinase-C, and nuclear factor erythroid 2-related factor (NFE2RF) pathways [76].

## 5. Conclusions

The inclusion of SP into the dietary ingredients of laying hens showed a significant improvement in the productive performance, cholesterol profile, redox status and inflammatory signs. There was a linear response in most parameters to increasing the SP level in layer diets, with the best results obtained when using SP at 90 g/kg in the diet. Exposure of laying hens to cyclic HS resulted in a significant increase in the total-CH, LDL-CH, liver- and egg yolk-CH, CP, MDA, IL-1β, IL6, and TNF-α, and a significant decrease in the HDL-CH, GSH, and TAC. When incorporating SP into the diets of HS-layers, the negative impacts of HS were remarkably relieved. Our results concluded that dietary inclusion of 90 g/kg SP could strongly contribute to increasing the protein contents of the diets compared to other traditional ingredients, and therefore, could be used as a promising nutritional approach to improving the performance, the physiology, and the health of laying hens, particularly under heat stress conditions.

## Figures and Tables

**Table 1:** The results of *Spirulina platensisis* (SP) chemical composition analysis.

Item	Contents in SP
Moisture (g) ^1^	5.6
Crude protein (g) ^1^	56.4
Total lipids (g) ^1^	7.2
Carbohydrate (g) ^1^	14.2
Crude fiber (g) ^1^	0.02
Total ash (g) ^1^	7.5
Energy (MJ) ^1^	43.6
Calcium (mg) ^1^	436.3
Phosphorus (mg) ^1^	124.5
Sodium (mg) ^1^	220.1
Potassium (mg) ^1^	167.8
Iron (mg) ^1^	11.5
Zinc (mg) ^1^	2.4
Total polyphenols (mg GAE/g) ^2^	22.1
Total flavonoids (mg QE/g) ^2^	6.7
Total antioxidant activity (%) ^3^	29.2

^1^ Calculated per 100 g SP. ^2^ Calculated as mg gallic acid equivalent (GAE) or quercetin equivalent (QE), respectively, per g dry weight of the SP. ^3^ Calculated as the percentage of the radical scavenging activity of the SP.

**Table 2:** Composition of the experimental diets ^1^.

Ingredients (g/kg as Fed)	0-SP	3-SP	6-SP	9-SP
Spirulina	0.0	30.0	60.0	90.0
Soybean meal (44% CP)	275.0	236.4	197.7	159.1
Yellow corn	566.5	575.1	583.8	592.4
Wheat bran	10.0	10.0	10.0	10.0
Soybean oil	30.0	30.0	30.0	30.0
Bone meal	30.0	30.0	30.0	30.0
Limestone	80.0	80.0	80.0	80.0
Salt (NaCl)	4.0	4.0	4.0	4.0
Premix ^2^	3.0	3.0	3.0	3.0
DL-Methionine	1.5	1.5	1.5	1.5
Nutrients ^3^				
Calculated metabolizable energy (MJ)	1.26	1.26	1.26	1.26
Calculated calcium (g)	40.2	40.2	40.2	40.2
Calculated available phosphorus (g)	5.2	5.2	5.2	5.2
Determined crude protein (g)	167.5	170.0	170.0	174.5
Determined crude fat (g)	66.0	64.5	63.8	62.1
Determined crude fiber (g)	47.0	46.5	46.5	45.8

^1^ Experimental diets: supplemented with 3% spirulina (3-SP), 6% spirulina (6-SP), 9% spirulina (9-SP), or without spirulina (0-SP). ^2^ Premix (content per kg of the experimental diet)): 8000 IU vitamin A; 1500 IU vitamin D; 4 mg riboflavin; 10 µg cobalamin; 15 mg vitamin E; 2 mg vitamin K; 500 mg choline; 25 mg niacin; 60 mg manganese; 50 mg zinc. ^3^ Calculated and determined nutrients were presented per kg of the diet.

**Table 3:** Effect of dietary *Spirulina platensisis* (SP) supplementation on the cholesterol profile of laying hens exposed to cyclic heat stress (HS).

Treatment Groups ^1^	HS (°C)	SP (%)	*n*	Total-CH, mg/dL	HDL-CH, mg/dL	LDL-CH, mg/dL	Yolk-CH, mg/dL *	Liver-CH, mg/dL *
HS	24	-	72	138.7 ^b^	56.3 ^a^	101.9 ^b^	12.2 ^b^	5.4 ^b^
	35	-	72	162.7 ^a^	45.2 ^b^	123.3 ^a^	21.2 ^a^	8.1 ^a^
SEM				0.38	0.34	0.50	0.17	0.19
SP	-	0	36	162.9 ^a^	45.9 ^c^	120.7 ^a^	18.9 ^a^	8.5 ^a^
	-	3	36	157.4 ^b^	47.5 ^c^	115.6 ^b^	16.9 ^b^	6.9 ^b^
	-	6	36	146.1 ^c^	52.4 ^b^	109.5 ^c^	15.5 ^c^	5.8 ^c^
	-	9	36	136.5 ^d^	57.3 ^a^	104.5 ^d^	15.6 ^c^	5.7 ^c^
SEM				0.54	0.48	0.71	0.24	0.27
HS × SP	24	0	18	152.3 ^d^	50.9 ^c^	113.2 ^d^	15.2 ^c^	6.9
	24	3	18	146.8 ^e^	52.6 ^c^	106.0 ^e^	12.5 ^d^	5.7
	24	6	18	135.5 ^f^	57.4 ^b^	98.0 ^f^	10.4 ^e^	4.7
	24	9	18	120.4 ^g^	64.3 ^a^	90.5 ^g^	10.8 ^e^	4.2
	35	0	18	173.5 ^a^	40.8 ^e^	128.3 ^a^	22.5 ^a^	10.2
	35	3	18	168.0 ^b^	42.3 ^e^	125.2 ^ab^	21.3 ^ab^	8.2
	35	6	18	156.7 ^c^	47.3 ^d^	121.0 ^bc^	20.6 ^b^	7.0
	35	9	18	152.6 ^d^	50.2 ^c^	118.5 ^c^	20.4 ^b^	7.2
SEM				0.76	0.68	1.00	0.34	0.39
*p*-value	HS			<0.001	<0.001	<0.001	<0.001	<0.001
	SP			<0.001	<0.001	<0.001	<0.001	<0.001
	HS × SP			<0.001	0.009	<0.001	<0.001	0.470
	SP-Linear contrast			<0.001	<0.001	<0.001	<0.001	<0.001
	SP-Quadratic contrast			<0.001	0.001	0.941	<0.001	0.007

Means with different superscripts in the same column, within main effect, indicate significant differences (*p* < 0.05), whereas means with the same or no superscripts indicate no significant differences (*p* > 0.05). ^1^ Treatment groups: HS, layers were exposed to either thermoneutral temperature at 24 °C or heat stress at 35 °C; SP, layers were fed a soybean–corn diet substituted partially with 0, 3, 6, and 9% *Spirulina platensisis*; HS × SP, interaction between HS and SP groups. *n*: number of observations per group. SEM: standard error of the mean. CH, cholesterol; HDL, high density lipoprotein; LDL low density lipoprotein. * Values were calculated per dL of the extracted solution from 10 mg of the harvested tissue.

**Table 4:** Effect of dietary *Spirulina platensisis* (SP) supplementation on the redox status of laying hens exposed to cyclic heat stress (HS).

Treatment Groups ^1^	HS (°C)	SP (%)	*n*	CP, ng/mL	MDA, nM/mL	TAOC, U/mL	GSH, nM/mL
HS	24	-	72	1009.4 ^b^	2.1 ^b^	8.9 ^a^	30.8 ^a^
	35	-	72	1904.2 ^a^	4.0 ^a^	6.5 ^b^	24.0 ^b^
SEM				9.24	0.09	0.19	0.19
SP	-	0	36	1607.9 ^a^	3.9 ^a^	6.6 ^b^	23.8 ^d^
	-	3	36	1536.8 ^b^	3.4 ^b^	7.3 ^b^	26.0 ^c^
	-	6	36	1421.6 ^c^	2.7 ^c^	8.4 ^a^	28.5 ^b^
	-	9	36	1260.9 ^d^	2.1 ^d^	8.5 ^a^	31.4 ^a^
SEM				13.07	0.13	0.27	0.26
HS × SP	24	0	18	1057.7 ^e^	2.5 ^cd^	7.4 ^bc^	27.2 ^c^
	24	3	18	1037.5 ^ef^	2.3 ^cd^	8.1 ^b^	28.2 ^c^
	24	6	18	983.5 ^ef^	1.8 ^de^	10.1 ^a^	31.5 ^b^
	24	9	18	958.9 ^f^	1.5 ^e^	10.0 ^a^	36.1 ^a^
	35	0	18	2158.1 ^a^	5.3 ^a^	5.9 ^c^	20.4 ^f^
	35	3	18	2036.1 ^b^	4.6 ^a^	6.5 ^bc^	23.7 ^e^
	35	6	18	1859.8 ^c^	3.6 ^b^	6.6 ^bc^	25.5 ^d^
	35	9	18	1562.9 ^d^	2.7 ^c^	7.0 ^bc^	26.7 ^cd^
SEM				18.48	0.18	0.39	0.37
*p*-value	HS			<0.001	<0.001	<0.001	<0.001
	SP			<0.001	<0.001	<0.001	<0.001
	HS × SP			<0.001	<0.001	0.019	<0.001
	SP-Linear contrast			<0.001	<0.001	<0.001	<0.001
	SP-Quadratic contrast			0.001	0.496	0.290	0.179

Means with different superscripts in the same column, within main effect, indicate significant differences (*p* < 0.05), whereas means with the same or no superscripts indicate no significant differences (*p* > 0.05). ^1^ Treatment groups: HS, layers were exposed to either thermoneutral temperature at 24 °C or heat stress at 35 °C; SP, layers were fed a soybean–corn diet partially substituted with 0, 3, 6, and 9% *Spirulina platensisis*; HS × SP, interaction between HS and SP groups. *n*: number of observations per group. SEM: standard error of the mean. CP, ceruloplasmin; MDA, malondialdehyde; TAOC, total antioxidant capacity; GSH, reduced glutathione.

**Table 5:** Effect of dietary *Spirulina platensisis* (SP) supplementation on the inflammatory cytokines of laying hens exposed to cyclic heat stress (HS).

Treatment Groups ^1^	HS (°C)	SP (%)	*n*	IL-1β, pg/mL	IL-6, pg/mL	TNF-α, pg/mL
HS	24	-	72	241.9 ^b^	2.8 ^b^	96.5 ^b^
	35	-	72	588.7 ^a^	10.8 ^a^	141.8 ^a^
SEM				10.36	0.11	0.45
SP	-	0	36	531.6 ^a^	8.9 ^a^	128.7 ^a^
	-	3	36	458.4 ^b^	8.3 ^b^	121.5 ^b^
	-	6	36	376.6 ^c^	6.2 ^c^	115.0 ^c^
	-	9	36	294.7 ^d^	3.7 ^d^	111.6 ^d^
SEM				14.65	0.16	0.63
HS × SP	24	0	18	259.5 ^e^	3.7 ^d^	103.9 ^e^
	24	3	18	257.6 ^e^	2.6 ^e^	96.6 ^f^
	24	6	18	230.3 ^e^	2.6 ^e^	93.6 ^f,g^
	24	9	18	220.4 ^e^	2.1 ^e^	92.0 ^g^
	35	0	18	803.7 ^a^	14.1 ^a^	153.5 ^a^
	35	3	18	659.3 ^b^	13.9 ^a^	146.4 ^b^
	35	6	18	522.9 ^c^	9.7 ^b^	136.3 ^c^
	35	9	18	368.9 ^d^	5.4 ^c^	131.1 ^d^
SEM				20.72	0.22	0.89
*p*-value	HS			<0.001	<0.001	<0.001
	SP			<0.001	<0.001	<0.001
	HS × SP			<0.001	<0.001	<0.001
	SP-Linear contrast			<0.001	<0.001	<0.001
	SP-Quadratic contrast			0.766	<0.001	0.003

Means with different superscripts in the same column, within main effect, indicate significant differences (*p* < 0.05), whereas means with the same or no superscripts indicate no significant differences (*p* > 0.05). ^1^ Treatment groups: HS, layers were exposed to either thermoneutral temperature at 24 °C or heat stress at 35 °C; SP, layers were fed soybean–corn diet partially substituted with 0, 3, 6, and 9% *Spirulina platensisis*; HS × SP, interaction between HS and SP groups. *n*: number of observations per group. SEM: standard error of the mean. IL-1β, interleukin-1 beta; IL-6, interleukin-6; TNF-α, tumor necrosis factor-alpha.

**Table 6:** Effect of dietary *Spirulina platensisis* (SP) supplementation on the productive performance of laying hens exposed to cyclic heat stress (HS).

Treatment Groups ^1^	HS (°C)	SP (%)	*n*	EP, %	EW, g	FI, g	FCR
HS	24	-	144	90.5 ^a^	61.8 ^a^	111.6 ^a^	2.00 ^b^
	35	-	144	77.0 ^b^	56.8 ^b^	101.5 ^b^	2.37 ^a^
SEM				0.36	0.08	0.13	0.013
SP	-	0	72	78.8 ^c^	56.7 ^d^	103.3 ^d^	2.38 ^a^
	-	3	72	83.5 ^b^	57.8 ^c^	105.3 ^c^	2.21 ^b^
	-	6	72	85.1 ^b^	60.7 ^b^	107.6 ^b^	2.09 ^c^
	-	9	72	87.6 ^a^	61.9 ^a^	110.2 ^a^	2.04 ^c^
SEM				0.51	0.11	0.18	0.018
HS × SP	24	0	36	89.1 ^b^	61.1 ^b^	107.0 ^d^	1.96 ^d^
	24	3	36	88.9 ^b^	61.4 ^b^	109.7 ^c^	2.01 ^d^
	24	6	36	91.3 ^ab^	62.2 ^a^	113.9 ^b^	2.01 ^d^
	24	9	36	92.7 ^a^	62.3 ^a^	115.9 ^a^	2.01 ^d^
	35	0	36	68.6 ^e^	52.3 ^e^	99.5 ^g^	2.79 ^a^
	35	3	36	78.1 ^d^	54.3 ^d^	100.9 ^f^	2.42 ^b^
	35	6	36	78.9 ^d^	59.2 ^c^	101.3 ^f^	2.18 ^c^
	35	9	36	82.4 ^c^	61.4 ^b^	104.4 ^e^	2.07 ^c,d^
SEM				0.73	0.16	0.25	0.026
*p*-value	HS			<0.001	<0.001	<0.001	<0.001
	SP			<0.001	<0.001	<0.001	<0.001
	HS × SP			<0.001	<0.001	<0.001	<0.001
	SP-Linear contrast			<0.001	<0.001	<0.001	<0.001
	SP-Quadratic contrast			0.037	0.808	0.161	0.004

Means with different superscripts in the same column, within main effect, indicate significant differences (*p* < 0.05), whereas means with the same or no superscripts indicate no significant differences (*p* > 0.05). ^1^ Treatment groups: HS, layers were exposed to either thermoneutral temperature at 24 °C or heat stress at 35 °C; SP, layers were fed soybean–corn diet partially substituted with 0, 3, 6, and 9% *Spirulina platensisis*; HS × SP, interaction between HS and SP groups. *n*: number of observations per group. SEM: standard error of the mean. EP, hen–day egg production; EW, egg weight; FI, feed intake; FCR, feed conversion ratio.

## Data Availability

Not applicable.

## Supplementary Materials

The following supporting information can be downloaded at: https://www.mdpi.com/article/10.3390/ani12202759/s1, Figure S1: A scheme for the experimental design.
