# Supplementary figures and images for: Hypocholesterolemic, Antioxidative, and Anti-Inflammatory Effects of Dietary Spirulina platensisis Supplementation on Laying Hens Exposed to Cyclic Heat Stress
Source: Animals (Basel). 2022 Oct 14;12(20):2759. doi: 10.3390/ani12202759 (PMC9597838; doi:10.3390/ani12202759)

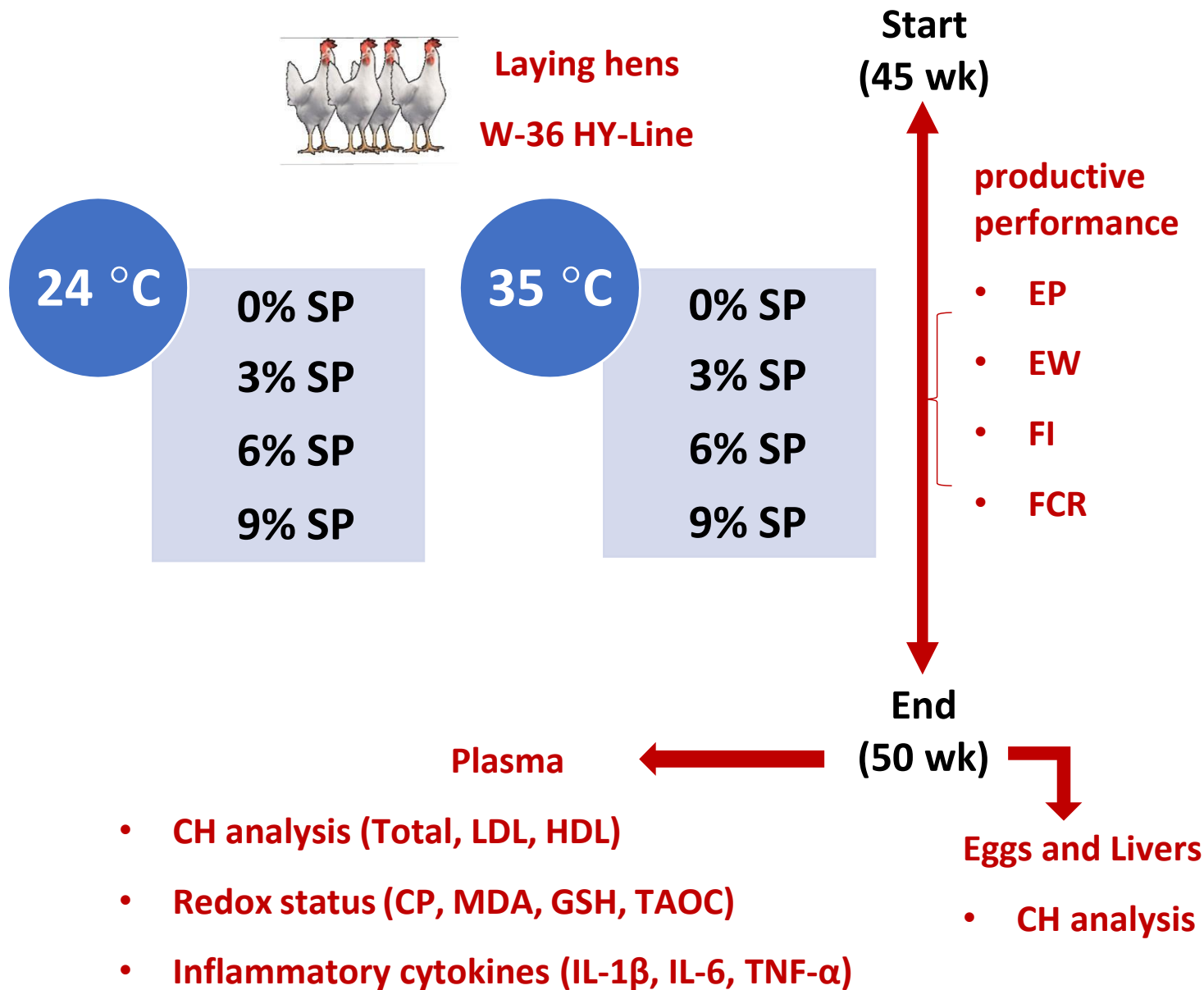

Figure S1: A scheme for the experimental design

Supplement: Supplementary file 1 [file animals-12-02759-s001.zip › animals-1950218-supplementary.pdf]
